# Evaluation of Prescriptions Dispensed in the Outpatient Pharmacies of a University Teaching Hospital in Moshi, Kilimanjaro Region, Tanzania: A Cross-Sectional Study

**DOI:** 10.24248/EAHRJ-D-18-00026

**Published:** 2019-11-29

**Authors:** Aristides B Audax, Eva P Muro

**Affiliations:** a Kilimanjaro Christian Medical University College, Moshi, Tanzania; b Department of Pharmacology, Kilimanjaro Christian Medical University College, Moshi, Tanzania; c Department of Pharmacy, Kilimanjaro Christian Medical Centre, Moshi, Tanzania

## Abstract

**Background:**

Prescriptions written in daily medical practice are associated with increasing numbers of prescription writing errors. Both omission and commission errors are encountered and caused by prescribers of different cadres. Prescribing errors are associated with adverse drug events (ADEs), which are harmful to patients. This study aimed to determine the common prescription errors, the categories of prescribers who commit prescription errors, and the most prescribed drugs in the outpatient pharmacies in general practice at Kilimanjaro Christian Medical Centre (KCMC).

**Methods:**

A prospective cross-sectional descriptive study was conducted at KCMC, a referral and teaching university hospital. All prescription dispensed on 2 June 2017 from the hospital's 2 outpatient pharmacies were reviewed, and our analysis determined the different types of prescription errors. A form designed by the authors was used to collect data from the prescription forms.

**Results:**

A total of 242 prescriptions were studied, and the most common omission errors were missing patients' weight (n=231, 95.5%), missing patients' address (n=213, 88.0%), missing drug dosage (n=159, 67.1%), and commission errors were due to wrong drug strength (n=10, 2.0%). Intern doctors were leading in writing prescriptions with errors (n=352, 25.6%), followed by residents (n=199, 14.5%), registrar doctors (n=167, 12.1%), and specialists (n=45, 3.3%). The most commonly prescribed drugs were antibiotics (n=120, 17.3%), antihypertensives (n=81, 11.7%), and analgesics (n=86, 12.4%).

**Conclusion:**

There were significant prescription errors at the study site, hence an intervention is needed to improve skills of prescribers. Educational interventions can substantially contribute to minimising such errors. Initaiting programmes and short courses on prescription writing before medical internship at the health facility might also be helpful.

## INTRODUCTION

Prescriptions are legal instructions written by medical practitioners that authorise patients to be issued medications by a pharmacist or dispenser. Prescriptions must be written correctly to ensure clear communication to to the pharmacist or dispenser. The normal features of a prescription to be legible the following parameters should be written, according to the World Health Organization (WHO): (a) prescriber information (hospital name, patient's address, information about the department and unit, plus details about the prescriber, such as name, designation and signature); (b) patient information (name, sex, age, weight, and address as well as the prescription date); (c) details of the medication prescribed (drug name, drug strength, and drug frequency of administration, the quantity that is to be dispensed, route of administration, drug dosage form and instructions about the use of the drug). Inappropriate prescription writing do not communicate clearly with the drug dispenser, as the results, there is occurrence of errors. An error is defined as something that is incorrectly done through either inadvertence or ignorance, or a mistake, for example, in judgment or calculation, speech, or even in writing action.^1^ These errors encountered in prescription writing are called prescription errors.

A prescription error is a failure in the prescription writing process that results in a wrong instruction about one or more features of a prescription. Prescription errors are mainly of 2 types. These are errors of omission and errors of commission. Errors of omission are due to incompleteness of the prescription form, that is there is missing information related to either the prescriber or patient or the drug. Lack of attention in prescription writing to ensure that all necessary information are filled properly leads to losing of skills, time and energies that is put during diagnosing a patients and deciding therapy.^2^ Errors of commission are due to wrong information about the prescribed drug; these regard wrong dosage form, wrong strength, and wrong drug name. Errors of commission such as wrong dose and wrong strength may result into serious effects or consequences since there are various forms and strengths of drugs available. This also results into either underuse of the prescribed drug or overuse of the drug prescribed.^3^

The occurrence of suboptimal prescribing such as under-dose, overdose and inappropriate use of drugs exists worldwide and has been associated with the increase in adverse drug reactions (ADRs) which are significant causes of drug related hospital admission.^4^ In addition, misuse and overuse of antibiotics could increase the duration of the disease and associated costs and increase resistance to infections as well as increase the risk of ADRs.^5^ Also, omission errors may cause the pharmacists, physicians as well as patients to waste a lot of time while the physician is called by the pharmacist to complete missing information or instructions.^2^

Prescribing errors are also associated with occurrence of adverse drug events (ADEs) of 26%–42% among patients and these ADEs can be prevented by simply avoiding prescription writing errors. The WHO definition of ADE is ‘any untoward medical occurrence that may present during treatment with a medicine, but which does not necessarily have a causal relationship’. Although a little is known on the effects or harm of some other factors like: route of drug administration, type of drug to the patients.^6^ The most common affected systems by these ADEs following errors in prescription writing are; the respiratory system, cardiovascular system, gastrointestinal system, central nervous system and the endocrine system.^7^

It has been shown that prescription writing errors are common and accounts for 70% of all medication errors which could result in occurrence of ADEs.^8^ A cross-sectional study that was performed at the university hospital in Shiraz, in Iran indicated that 96.5% of prescribed prescriptions contained prescription writing errors.^6^ Also in a retrospective cross-sectional study that was conducted at Tikur Anbessa Hospital in Ethiopia, the results were that 42.89% of the studied prescriptions were found to contain omission errors.^9^ This shows that prescription writing errors are more to be encountered in various regions involving prescribing practice.

In Tanzania, a descriptive study conducted in various private hospitals in Dar es Salaam showed that of all prescriptions studied, each prescription had at least more than one error in 99.6%.^10^ Due to these high occurrences of prescription errors, there are different interventions that have been done to minimise prescription errors. In developed countries, a system of electronic prescription has been adopted and studies have shown that prescription writing errors can be minimised. A study on evaluation of the system of electronic prescribing concluded that: among 25 studies that were carried out, 23 showed a significant reduction in relative risk ranging from 13% up to 99%. Thus, it is seen that electronic system used in prescribing can lead to reduction of the risk of committing prescription errors.^11^

Since prescription writing errors can be avoided, it is a important target for improvement so as to decrease medication errors and their undesirable effects.^11,12^ But also considering that prescription order in our areas being hand written, there is a high susceptibility of committing errors in the process of prescription writing, but also due to the absence of any supporting system to aid prescribers, who rely mostly on their memory in order to avoid prescribing errors.^9^ Few epidemiological statistics have been put forward with regard to common prescription writing errors in the developing countries.^9^

In Tanzania, studies have shown also that prescription writing errors are commonly encountered8. This study showed that 99.6% of all prescriptions had at least one or more errors which involved omission of either the patient's age (2.9%), name (1.6%), weight (93.8%) or route of administration (94%), dose (5.4%), frequency (3.2%), dosage form (24.8%) and duration of treatment (14.1%). Errors of commission accounted for 3.1% of all prescribed medicine.^10^ An other study done in Mwanza showed that among all prescriptions observed, 485 (85.1%) had at least one or more errors.^13^ The lack of self-awareness of errors among prescribers contributes to prescription writing errors. KCMC being one of the medical institutions serving more than 15 million Tanzanians, still a little is known about prescription writing errors at KCMC. KCMC is a referral hospital for over 15 million people in Northern Tanzania. The hospital is a huge complex with 500–800 inpatients in 630 official beds and 90 canvas and 800–1000 outpatients daily. The capacity of pharmacy to store and dispense drugs was 75–80% according to their reports.

Therefore, the objective of the present study was to determine the common prescription writing errors and prescribers who commit prescription writing errors at KCMC. The study will also find out the most prescribed drug at KCMC outpatient pharmacies.

## METHODS

### Study Design

This was a prospective cross-sectional descriptive study that was carried out at the Department of Pharmacy outlets for outpatient clinic at KCMC a teaching and consultant hospital, after obtaining Institutional Ethical Clearance. The prescription writing errors such as errors of omission related to prescriber (Patients name, Age, O/P Number, Date, Prescribers name, Prescribers signature, Clinic/Department, Weight, Diagnosis and illegible prescriptions); errors of omission related to drugs (Route of administration, Dose, Frequency, Strength, Dosage form, Duration/number of doses, Quantity to supply) and Errors of commission (Wrong strength, Wrong dosage form, Wrong drug name) were documented in a designed documentation form.

### Study Site

The study was conducted at Kilimanjaro Christian Medical Centre located in the foothills of the snow-capped, Mount Kilimanjaro, Tanzania. It is a referral consultant and teaching hospital with outpatient clinic serving a population of more than 15 million in northern Tanzanian, attending more than 800–1000 patients daily at the outpatient department. Thus, the area was selected for this study because of its accessibility and availability of data, and also considering the research timeframe. The pharmacy service is also divided into outpatient and inpatients pharmacies known as satellite pharmacies. The study was conducted in the outpatient pharmacy.

### Data Collection Tools

A check list was used to extract data from the prescription forms. All outpatient prescriptions that were prescribed and dispensed on 2 June 2017 were used for data collection because all the outpatient clinics were running on that day. The data collection tool was designed to indicate all information that should be filled on the prescription form. Also, a checklist contained addition columns for recording wrong drug information (ie, wrong dosage, wrong strength, wrong frequency).

### Data Collection Method

On 2 June 2017, prescription forms were obtained from the KCMC outpatient pharmacy where patients were attending to buy the prescribed drugs. The prescription form was recorded immediately after the patient handled the prescription form to the pharmacist/dispenser. But this was done between a researcher and a pharmacist/dispenser without involving the patient. Each legible prescription was recorded. After recording a corresponding prescription form, it was handled back to the dispenser to continue with his/her services.

The following variables were collected to determine errors of omission: prescriber's signature, code, date of prescription writing, patient's name, age, weight, address, and drug name, strength, and frequency of administration, quantity to be dispensed, route, and dosage form were checked if they have been filled on the prescription form, and they were indicated by ✓ sign for the filled information, and an X sign for the missing information on a form designed by the author.

Furthermore, the prescriptions were reviewed to check if they contained wrong dosage form, wrong strength, wrong frequency of administration and wrong drug name to determine the errors of commission. Also all the prescribed drugs were recorded in order to determine the most prescribed drugs.

### Data Analysis

The data collected by the author's themselves were entered and analysed in Social Package for Social Science (SPSS) version 20(SPSS Inc., Chicago, IL, USA). Descriptive analysis was conducted, and all the results were expressed as frequencies and percentages. Categorical data were summarised using percentages and were presented using narration and tables.

### Ethical Approval

Permission to conduct the study was gained from Research and Ethics Committee of the Kilimanjaro Christian Medical University College. A permit to access the prescription forms was requested from the Executive Director of the Kilimanjaro Christian Medical Centre. Neither the real names of prescribers nor the departments were in the report; this was for ensuring confidentiality.

## RESULTS

A total of 581 medicines were prescribed in the 242 prescriptions, giving an average of 3.8 drugs per prescription that were collected at Kilimanjaro Christian Medical Centre out-patient pharmacies on the 2 June 2017.

### Prescription Errors

A total of 242 prescriptions were collected analysed, out of which 933 prescription writing errors were noted with an average of 3.90 errors per each prescription, among them 923 errors of omission and 10 0f omission errors (Table 1).

#### Errors of Omission

Among 923 omission errors, 610 omissions errors related to prescriber (3.8 errors per each prescription) were found (Table 1). These were due to failure in mentioning the patient's weight (n=231, 95.5%), followed by patient's address (n=213, 88.0%), diagnosis (n=99, 41.1%), patient's age (n=27, 3.9%), prescriber code (n=19, 7.9%), patient registration number (n=13, 5.4%), date (n=4, 1.7%), prescriber's signature (n=3, 1.24%), and patient's name (n=1, 0.4%) respectively (Table 2). Additionally, 313 omission errors related to drugs (1.3 errors per each prescription) were found being due to missing drug dosages (n=159, 67.1%), route of administration (n=110, 46.4%), strength (n=24, 10.1%), duration (n=11, 4.6%) and frequency (n=9, 3.8%) respectively (Table 3).

**TABLE 1.** Prescription Errors (N=933)

Type of Error	n	Errors per Prescription
**Errors of omission**	923	3.8
Prescriber-related	610	2.5
Drug-related	313	1.3
**Errors of commission**	10	0.04

**TABLE 2.** Prescriber-Related Errors of Omission

Missing Details	n (%)
Prescriber signature	3 (0.5)
Prescriber code	19 (3.1)
Date	4 (0.7)
Patient name	1 (0.2)
Patient address	213 (34.9)
Patient registration number	13 (2.1)
Patient age	27 (4.4)
Patient weight	231 (37.9)
Diagnosis	99 (16.2)
**Total**	**610**

**TABLE 3.** Drug-Related Errors of Omission

Missing Details	n (%)
Dose	159 (67.1)
Strength	24 (10.1)
Frequency	9 (3.8)
Duration	11 (4.6)
Route of administration	110 (46.4)
**Total**	**313**

#### Errors of Commission

A total of 10 errors of commission were reported with an average of 0.04 errors per prescription. These commission errors were due to wrong drug strength (n=10, 2%) (Table 4).

**TABLE 4.** Errors of Commission

Error Description	n (%)
Wrong drug dose	0 (0.0)
Wrong drug strength	10 (100)
Wrong drug name	0 (0.0)
Overlooked drug–drug interaction	0 (0.0)
**Total**	**10**

### Prescribers Who Commit Writing Errors

Among 933 errors that were found in 242 prescriptions, a total of 352 (25.6%) errors were found on prescriptions written by intern doctors, residents (n=199, 14.5%), and registrar doctors (n=167, 12.1%), respectively. A total of 105 (8%) errors were found on prescriptions which did not contain prescrib er's code, 45 (3.4%) errors were on prescriptions written by specialists, 40 (2.9%) errors on prescriptions by dermatology special course doctors, and 15 (1.1%) errors were found on prescriptions written by assistant medical officers (Table 5).

**TABLE 5.** Error Frequency by Prescriber Position

Prescriber Position	n (%)
Specialist	45 (3.3)
Registrar	167 (12.1)
Intern	352 (25.6)
Resident	199 (14.5)
Dermatology special course trainee	40 (2.9)
Assistant medical officer	15 (1.1)
Unknown	105 (7.6)
**Total**	**923**

### The Most Commonly Prescribed Drugs

Total number of drugs prescribed was 581, among them 120 (21.4%) prescriptions were for antibiotics, 81 (11.7%) were for antihypertensives, 86 (12.4%) were for analgesics, 30 (4.5%) were for anticoagulants, 34 (4.9%) were for antihistamines, 22 (3.2%) were for cough suppressants, and 38 (5.5%) were for minerals, respectively (Table 6).

**TABLE 6.** Most Commonly Prescribed Drugs

Drug Category	n (%)
Analgesics	86 (15.2)
Antihypertensives	81 (11.7)
Anitbiotics	120 (17.3)
Antihistamines	34 (4.9)
Cough suppressants	22 (3.2)
Minerals	38 (5.5)
Others	169 (24.4)
**Total**	**550**

## DISCUSSION

In the present study, we revealed that there was an average of 3.9 errors per prescription among the 242 prescriptions coming from the different dispensing outlets at the hospital pharmacies, All the prescriptions (100%) had one or other prescription writing error. There were 10 commission errors that were found (2%), all of them regarding to wrong strength of the medicine. Whereas a study conducted in Malaysia by Kuan Mun Ni et al reported 96.7%, and Muyogela et al reported 99.6% of all prescription writing errors. Among 923 errors of omission, 610 of omission were related to prescriber (3.8 errors per prescription) were reported and these were due to failure to mention patient weight (95.5%) followed by failure to mention the address of patient (88.0%) failure to mention the diagnosis (41.1%), age (3.9%), name (0.40%), route of administration (46.4%), dosage form (67.1%).

A similar study conducted in Dar es salaam, Tanzania, by Mugoyela et al^10^ reported that errors which involved omission of either patients names not mentioned was 1.6% and weight of patients not mentioned in 93.8% of all prescriptions.

A study conducted by Kuan Mun Ni et al reported 397 errors of omission related to prescriber and were due to failure to mention age (32.7%) followed by date (17.1%), clinic or department (16.4%), registration number (0.5%), prescriber's name (1.8%), prescriber's signature (0.3%) and illegible hand writing (7.1%) and 862 errors of omission were due to failure to mention route of administration (80%), strength (56.3%), dosage form (36.4%), duration or number of doses (8.8%), dose (8.7%), quantity to supply (5.8%), frequency (5.3%) and drug name (0.2%).

Our study reveals prescription (100%) had one or other prescription writing errors slightly more when compared to Kuan Mun Ni et al (96.7%) and Mugoyela et al (99.6%). Similar results were also shown by a study conducted in Mumbai, India which found that patients’ names were mentioned in 94.75% while weight was mentioned on only 10% of all prescriptions.^3^

These results are contrary from results of a study that was carried out in a teaching hospital in India where they found that patients’ weight was not mentioned in only 8.18% of all 220 prescriptions. Also it was found that there were errors of commission regarding to wrong dosage 21.36%, wrong strength 5.90% and wrong drug name 5%.^2^ The difference may have been due to the fact that more than 90% of prescriptions that were written on 2ndJune, 2017 when this study was conducted were for adults, and so, clinicians/prescribers do not use body weights since most drug strengths for adults are not calculated per kilogram body weight as in paediatrics. Among all errors that were found in this study, only 3.3% of all errors were caused by specialists while registrar doctors, residents and Interns caused 12.5%, 14.5%, 25.6% of all errors respectively. The most errors committed being errors of omission whereby Intern doctors omitted dosage, patient's weight and address in 81.8%, 98% and 86% respectively, residents mostly omitted patient's address and patients weight in 80.4% and 85.7% respectively and registrar doctors mostly omitted patient's address in 97.6% of the prescriptions that they wrote. These results show that Intern doctors are leading in writing prescriptions with errors; these results are similar to other studies that have shown that causing prescription writing errors among registrar doctors and residents^14^ also similar to another study which showed that interns and registrar doctors are more likely to cause errors than specialists.^2^

In this study, a total 581 medicines were prescribed and among them, the most prescribed medicines were antibiotics (17.3%) of all prescribed medicines followed by antihypertensive (11.7%), analgesics (12.4%), minerals (5.5%) and antihistamines (4.9%). Comparative results were found in a study conducted in Nigeria which showed that analgesics (19.7%) were leading followed by antibiotics (13.0%) and antihypertensive (4.9)^15^ and another study showed that analgesics were prescribed in 25.78%, antibiotics in (22.1%), antihistamines in (6.25%).^16^ In this study, higher percentages of antihypertensives were encountered on the prescriptions as compared to other studies. This may be due to the fact that this study was carried out on Friday 2 June 2017, during which the scheduled hypertension clinic was held. The number of high prescription of antibiotics observed in this study might be due to irrational prescribing practice among prescribers. Also, empirical prescribing of antibiotics without microbiological diagnostic sensitivity testing due to lack of these tests and hospital antibiotic policy. This practice may have promoted higher use of antibiotics, which ultimately leads to development of bacterial resistance and increases the necessity to use expensive antibiotics.

### Limitations

This study was cross-sectional hospital-based study that was conducted for only one day rather than months as for other studies due limited budget and time with a small sample size of which the result limits generalisation to the population.

## CONCLUSION AND RECOMMENDATIONS

The results of this study indicated the presence of significant prescription errors at the study site which highlights a need of conducting educational programmes among all prescribers especially interns in order to improve their skills of prescription writing. An emphasis being put on avoiding the most committed errors of omission that is patient's address, dosage and route of administration of the prescribed medicines. In turn this will help to minimise medication errors that are due to prescription errors. There must be a control of prescribing antibiotics by limiting the level of prescriber since in this study 41.5% of all prescribed antibiotics were prescribed by intern doctors. Further studies are warranted to find out what factors cause prescribers to write prescription with errors; that is either missing information or wrong information.
